# Infectious Myositis of the Left Foot in a Healthy Adult Male

**DOI:** 10.7759/cureus.111548

**Published:** 2026-06-26

**Authors:** Hira Sheheryar, Madhura Vadlamudi-Chandrashekar, Muhammad Zakariyya Sharief, Mohiuddin Sharief

**Affiliations:** 1 Acute Medicine, Lancashire Teaching Hospitals NHS Foundation Trust, Royal Preston Hospital (RPH), Preston, GBR; 2 Acute Medicine, School of Medical Sciences, University of Manchester, Manchester, GBR

**Keywords:** cellulitis mimic, focal myositis, foot infection, infectious myositis, necrotizing fasciitis differential

## Abstract

We report the case of a 37-year-old male who presented with non-traumatic pain and swelling of the left foot, initially managed as cellulitis with oral antibiotics. Due to progressive symptoms and rising inflammatory markers, he was admitted for further evaluation. MRI confirmed infectious myositis involving the deep intrinsic muscles of the foot, without abscess or necrotizing fasciitis. He responded well to intravenous and subsequently oral antibiotics, avoiding surgical intervention. This case underscores the need for early imaging in atypical or non-resolving limb infections and highlights the multidisciplinary approach in managing deep soft tissue infections.

## Introduction

Infectious myositis is a rare but potentially serious condition involving bacterial infection of skeletal muscle [1-5]. It often presents similarly to cellulitis or compartment syndrome, posing diagnostic challenges [6]. Early recognition and differentiation from necrotizing fasciitis or autoimmune myositis are crucial, as management strategies differ significantly [3-5]. The intrinsic muscles of the foot are anatomically complex and located within deep plantar compartments, making early clinical recognition of infection challenging. *Staphylococcus aureus* remains the most commonly reported causative organism in bacterial infectious myositis and pyomyositis, although blood cultures may frequently remain negative [6]. Unlike pyomyositis with abscess formation, localized infectious myositis may initially present without drainable collections, contributing to delayed diagnosis. MRI is the gold standard for detecting deep muscle infections and differentiating from urgent muscle disorders [7]. This report presents a case of isolated infectious myositis in a previously healthy adult, illustrating the importance of MRI and multidisciplinary collaboration [2-4].

## Case presentation

A 37-year-old man with a history of anxiety and depression presented with worsening left foot pain, swelling, and erythema following overcompensation due to a prior right great toe fracture. It was hypothesized that altered weight-bearing and repetitive compensatory stress may have resulted in minor muscular strain or microtrauma, potentially predisposing the affected musculature to localized infection despite the absence of penetrating injury. Initially treated as cellulitis with oral flucloxacillin, his symptoms progressed despite antibiotics, prompting hospital admission. Imaging revealed myositis of the deep intrinsic muscles of the left foot without evidence of necrotizing fasciitis or compartment syndrome [1-2]. Blood cultures remained negative, and no tissue aspiration or biopsy was performed as MRI demonstrated no drainable collection and the patient showed progressive clinical improvement with antimicrobial therapy. Therefore, the diagnosis was established radiologically in conjunction with clinical response to treatment.

**Figure 1:**
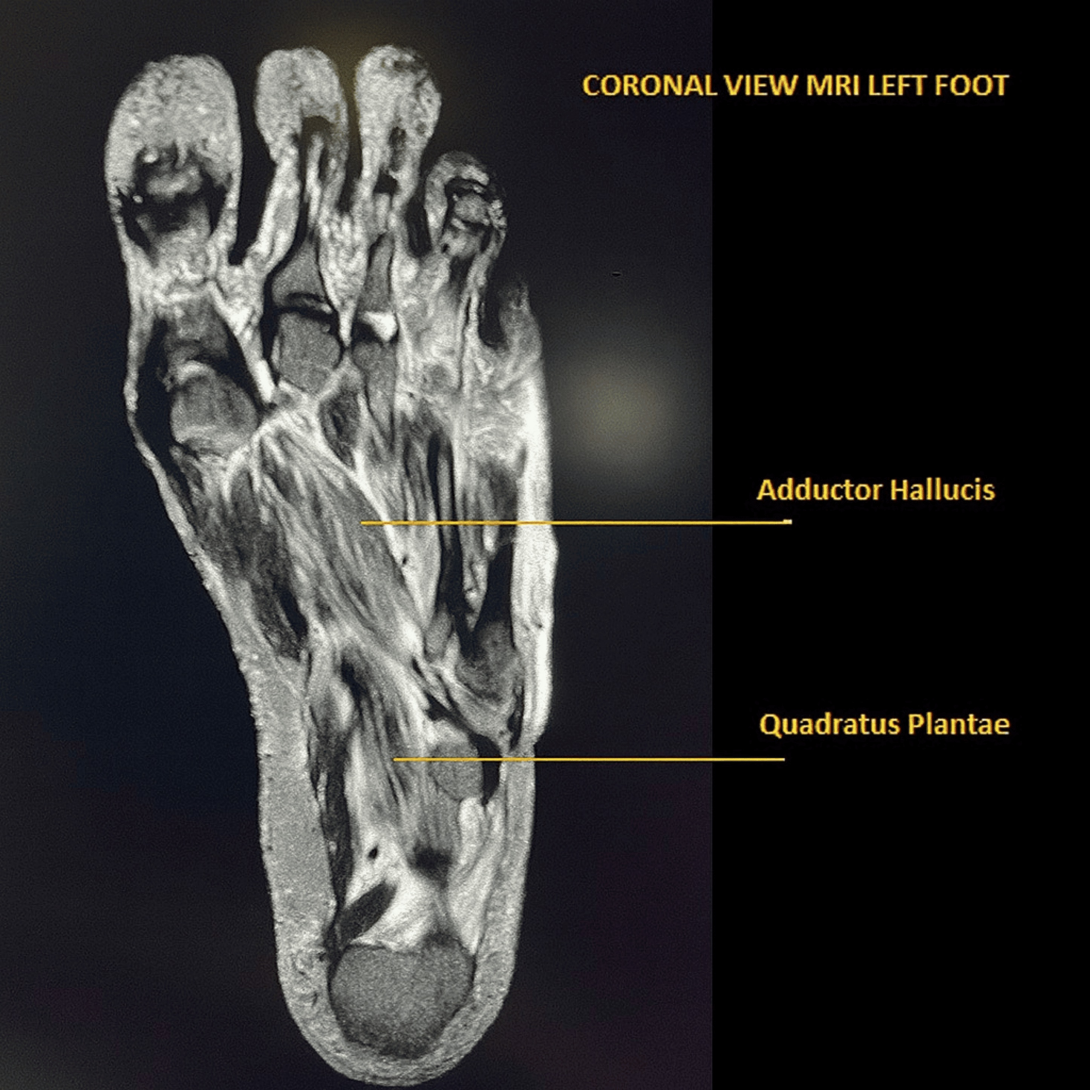
Coronal view MRI of left foot showing hyperintensity in the adductor hallucis and quadratus plantae muscles

**Figure 2:**
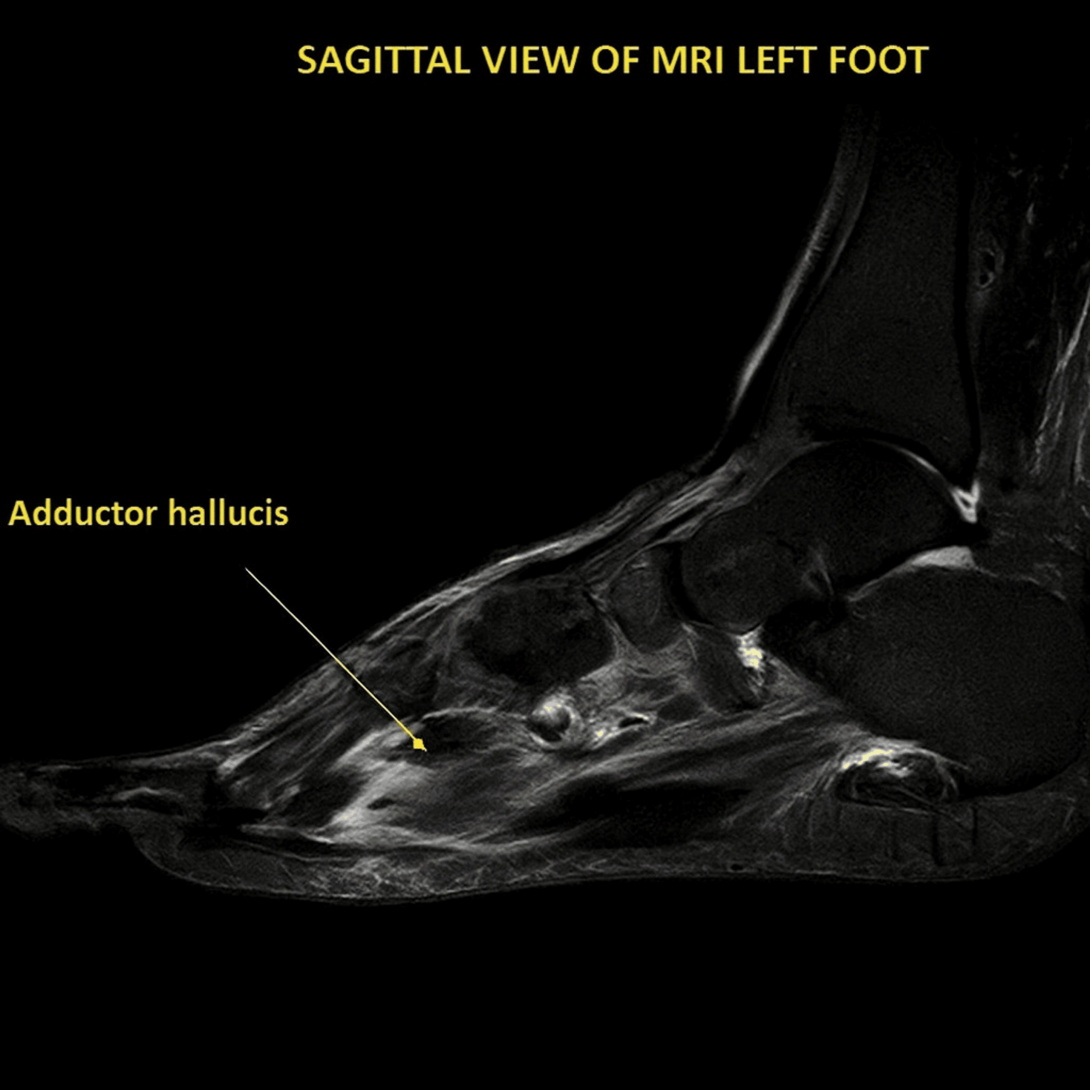
Sagittal STIR MRI of left foot STIR: short-TI inversion recovery.

He was managed with intravenous flucloxacillin and clindamycin, analgesia, and supportive care including elevation and mobilization aids. Over the course of admission, inflammatory markers gradually improved, and antibiotics were de-escalated to oral therapy. Rheumatology input concluded the myositis was infectious rather than autoimmune, and the patient was discharged with ongoing oral antibiotics and no need for further immunological screening [5-6]. Investigations have been highlighted in a summary table with reference values (Table 1). Magnetic resonance imaging of the left foot is shown in Figures 1, 2.

**Table 1:** Investigations NF: necrotizing fasciitis.

Investigations	Results	Reference Range
White blood cells (WBC)	14.42 x10⁹/L	4-11 ×10⁹/L
Neutrophils	11.07 x10⁹/L	1.60-7. 5 x10⁹/L
C-reactive protein (CRP)	332.3 mg/L (peak)	<5 mg/L
Procalcitonin (PCT)	1.47 ng/mL (peak)	0-0.24 ng/mL
Blood cultures	No growth	
X-ray left foot	No bony abnormality	
Magnetic resonance imaging of left foot	Myositis of deep intrinsic muscles; no abscess or NF	

Differential diagnosis

The differential diagnoses considered for this clinical presentation included cellulitis, which was initially suspected and subsequently supported by imaging demonstrating superficial soft tissue involvement. Necrotizing fasciitis was also considered; however, this was deemed unlikely following review by the Plastic Surgery team (Laboratory Risk Indicator for Necrotizing Fasciitis (LRINEC) score of 4 and a negative finger sweep test) [3,8]. Compartment syndrome was excluded following serial orthopaedic assessments demonstrating preserved neurovascular status, clinically soft compartments, and MRI findings without features suggestive of compartment syndrome. Compartment pressure measurements were therefore not deemed necessary [6]. Autoimmune myositis was also considered; however, there were no associated systemic features or supportive serological findings, and as patient was responsive to antibiotics, no further autoimmune workup was indicated [5].

Treatment

Initial oral therapy consisted of flucloxacillin 1 g QDS, which was escalated to intravenous flucloxacillin and clindamycin, and subsequently switched to IV tazocin and clindamycin due to persistent severe symptoms and markedly elevated inflammatory markers despite initial therapy, with the intention of providing broader antimicrobial and anaerobic coverage for possible deep soft tissue infection. After MRI confirmation of the diagnosis, intravenous antibiotics were continued before stepping down to oral flucloxacillin and clindamycin. The duration of therapy was guided by sustained clinical improvement, downward trends in inflammatory markers, absence of abscess formation on MRI, and multidisciplinary microbiology input. Analgesic management included IV morphine with transition to oral morphine and PRN paracetamol. Supportive measures involved leg elevation, walking aids (frame and crutches), physiotherapy, and input from the pain team. The management was based on continuous input from multidisciplinary team specialists from microbiology, orthopaedics, plastics, rheumatology, pain team, and physiotherapy.

Outcome and follow-up

At the time of discharge, the patient demonstrated improving mobility and weight-bearing ability with the assistance of walking aids. No focal neurological deficit or significant residual weakness was identified, and continued physiotherapy with gradual return to activity was advised. The patient showed gradual clinical improvement with resolution of erythema, decreased CRP (from 332.3 to 85.6 mg/L), and improved mobility. He was safely discharged with walking aids, appropriate oral antibiotics, and no need for ongoing rheumatology follow-up [2,5].

## Discussion

Infective myositis, an uncommon group of inflammatory myopathies caused by a wide range of infective agents, such as viral, bacterial, fungal, and parasitic, is a potentially treatable entity [9]. It is most often seen in young adults. Pyomyositis, or bacterial myositis, was once considered a tropical disease but is now seen in temperate climates, particularly with the emergence of HIV infection. Other risk factors for infectious myositis include: Rhabdomyolysis, muscle trauma overlying cellulitis, infected insect bites, injection of illicit drugs, and diabetes mellitus [10]. Clinically, infective myositis may display an acute, subacute, or chronic course marked by pain, tenderness, swelling, and /or weakness [9].

In this case, cellulitis was initially considered the working diagnosis; however, the degree of pain reported was disproportionate to the expected severity of superficial soft tissue inflammation. This discrepancy prompted consideration of more serious alternative diagnoses, including myositis, compartment syndrome, and necrotizing fasciitis. Necrotizing fasciitis (NF) is a rapidly progressive, life-threatening illness that's characterized by necrosis and suppuration. It is often difficult to differentiate NF from cellulitis or pyomyositis (PM) according to the initial clinical presentation [11]. However, following specialist input from the Plastic Surgery and Orthopaedic teams, necrotizing fasciitis and compartment syndrome were considered unlikely and effectively excluded. Autoimmune myositis was also considered; however, this was deemed unlikely given negative serological investigations and the patient’s clinical improvement with antibiotic therapy. Additionally, the elevated procalcitonin level further supported an underlying bacterial infective process and helped differentiate infectious myositis from autoimmune inflammatory myopathies or non-infectious muscle inflammation.

**Figure 3:**
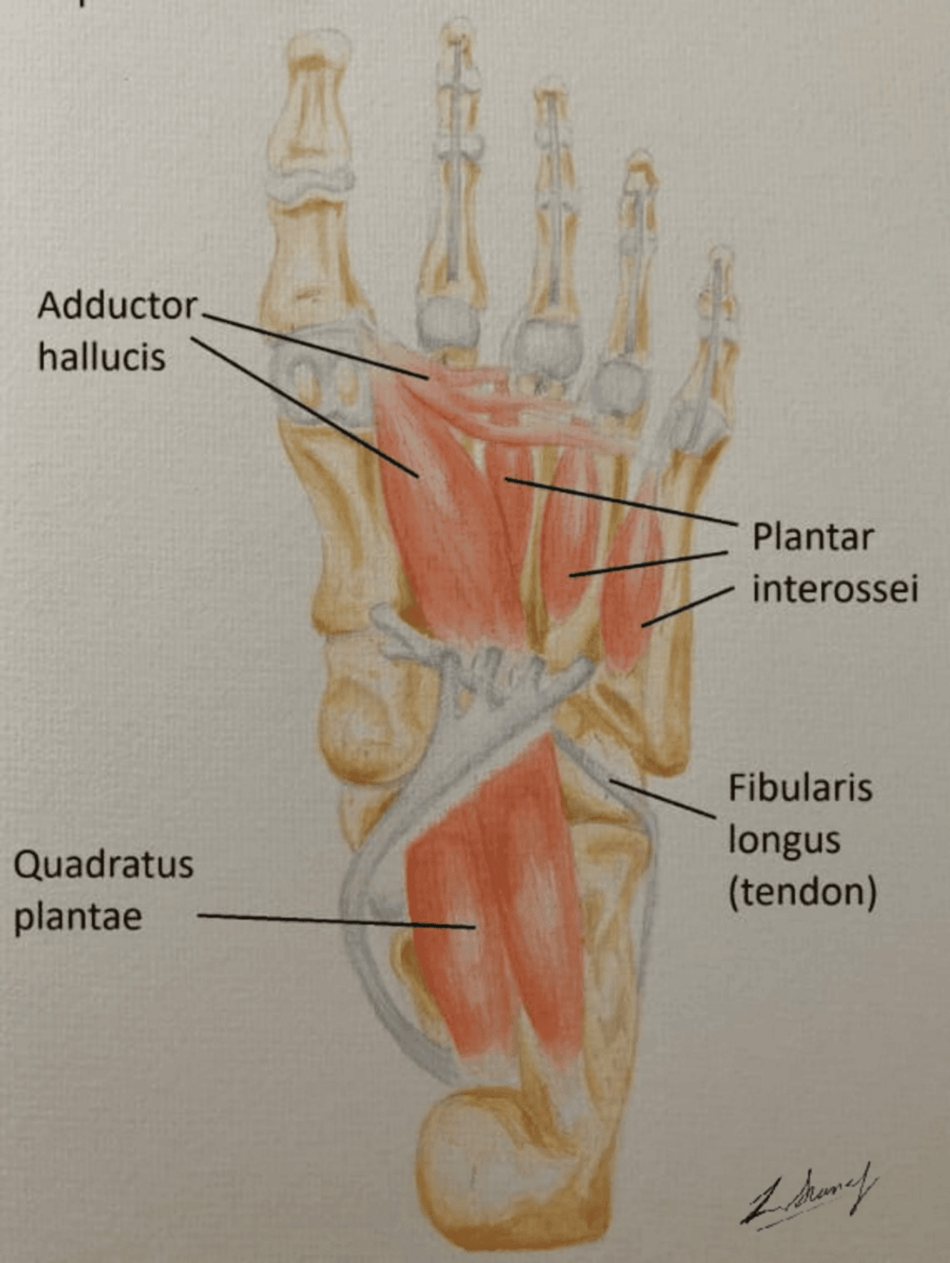
Anatomical illustration demonstrating the deep intrinsic plantar muscles involved in this patient’s infectious myositis Original illustration created by third author Muhammad Zakariya Sharief for this manuscript, adapted from a public domain Gray's anatomy image 444 [12].

Further investigations included MRI imaging to better characterize the extent of soft tissue involvement. MRI is the modality of choice to accurately assess the extent of involvement. Muscle oedema, characterised by a high T2 signal, is typically present. Abscesses appear, as elsewhere, as fluid collections (high T2, low T1) with peripheral contrast enhancement. There may also be diffuse muscle enlargement [10]. MRI imaging confirmed myositis, demonstrating involvement of the central deep plantar intrinsic muscles of the left foot, including the adductor hallucis, lumbricals, and quadratus plantae muscles, with probable mild involvement of the plantar interosseous muscles (Figure 3, indicating muscles involved). There was no evidence of rim-enhancing fluid collections or abscess formation requiring surgical drainage, supporting conservative medical management. Based on these findings, a management plan was formulated, incorporating both supportive measures and pharmacological treatment. A prompt and early treatment with appropriate antibiotics is necessary for complete recovery, while an incomplete or delay in treatment may cause complications that include sepsis [9].

## Conclusions

Infectious myositis involving the intrinsic muscles of the foot is uncommon and may occur even in the absence of penetrating trauma or immunocompromise. Clinicians should maintain a high index of suspicion in patients presenting with progressive foot pain and swelling that are disproportionate to superficial cellulitis or fail to respond to standard antibiotic therapy. MRI remains essential for identifying deep soft tissue involvement and excluding surgical emergencies such as necrotizing fasciitis or compartment syndrome. Early multidisciplinary management can facilitate successful non-operative treatment, functional recovery, and avoidance of unnecessary surgical intervention.
